# Epigenetic Remodeling in Male Germline Development

**DOI:** 10.1155/2016/3152173

**Published:** 2016-10-13

**Authors:** Na Li, Qiaoyan Shen, Jinlian Hua

**Affiliations:** College of Veterinary Medicine, Shaanxi Center of Stem Cells Engineering & Technology, Northwest A & F University, Yangling, Shaanxi, China

## Abstract

In mammals, germ cells guarantee the inheritance of genetic and epigenetic information across generations and are the origin of a new organism. During embryo development, the blastocyst is formed in the early stage, is comprised of an inner cell mass which is pluripotent, and could give rise to the embryonic stem cells (ESCs). The inner cell mass undergoes demethylation processes and will reestablish a methylated state that is similar to that of somatic cells later in epiblast stage. Primordial germ cells (PGCs) will be formed very soon and accompanied by the process of genome-wide demethylation. With the input of male sex determination genes, spermatogonial stem cells (SSCs) are generated and undergo the process of spermatogenesis. Spermatogenesis is a delicately regulated process in which various regulations are launched to guarantee normal mitosis and meiosis in SSCs. During all these processes, especially during spermatid development, DNA methylation profile and histone modifications are of crucial importance. In this review, we will discuss the epigenetic modifications from zygote formation to mature sperm generation and their significance to these development processes.

## 1. Introduction

Epigenetic modifications change dynamically during the process of germ cell development. In zygotes before the first round of mitotic division, genomes of both maternal and paternal source undergo robust active and passive demethylation [1]. Thereafter, during mitosis, the methylation patterns continue to change, from the preimplantation embryo ultimately to blastocyst composed of trophoblast and inner cell mass cells. The inner cell mass, which is the derivative source of embryonic stem cells, lies inside the blastocyst at embryonic day 3.5 (E3.5) in mice. Before implantation, this time point exhibits the lowest methylation level [2]. Thereafter, during the process of embryonic development, the epiblast shows global remethylation rapidly at E5.5. At E5.5, the activities of DNA methyltransferases DNMT3a and DNMT3b are active, contributing to rapid genome-wide DNA methylation. During this period, DNA methylation is targeted to germline genes to a large extent, and loss of suitable methylation would result in activation of specific genes in the embryo [3]. The genome-wide epigenetic states in PGC undergo extensive reprogramming to wipe DNA methylation, thus guaranteeing the two sexes acquire an equivalent epigenetic state [4]. Then, in the male mouse, sex-specific epigenetic patterns are reestablished, beginning before birth (E15.5–18.5) in prospermatogonia and complete remethylation at the termination of meiotic pachytene after birth (D10–19) [5].

In this review, we summarize the current knowledge about the epigenetic modifications during the differentiation process of ESC to epiblast, to PGC, to SSC, and at last to the process of spermatogenesis.

## 2. Embryonic Stem Cells

Embryonic stem cells (ESCs) are pluripotent stem cells which can self-renew indefinitely [6]. They are able to differentiate into all three germ layers under differentiation stimuli [7, 8]. In the maintaining of pluripotency, regulations from transcriptional factors to epigenetic modifications are both crucial [9].

### 2.1. Molecular Control of ESCs

The transcriptional factors OCT4, SOX2, and NANOG are the three most important transcriptional factors in ESC pluripotency maintenance, and they could both function individually and form a triumvirate to constitute a complicated regulatory network [10–12]. OCT4 is a well-accepted pluripotency factor, playing a dominant role in pluripotency maintenance; SOX2 is also essential to retain the maximum pluripotency capacity of the ESCs [13]. OCT4 and SOX2 begin to show high expression levels in the inner cell mass, but their levels decreased as the cells enter epiblast stage. Unlike OCT4 and SOX2, NANOG is highly expressed in the inner cell mass as well as in the epiblast cells of the embryo. NANOG deficient embryos fail to develop, but ESCs isolated from such embryos could be derived* in vitro*, indicating that NANOG is important for the regulation of cell fate at the early stages of development, even though they are not necessary for self-renewal [14].

### 2.2. Epigenetic Regulation of ESCs

Epigenetic regulations are usually associated with pluripotency states of ESCs. It was demonstrated that the ground state of naïve pluripotency of mouse ESCs (cultured in 2i LIF) led to low DNA methylation level [15–17], a flat pattern of H3K27me3 around transcription start sites and a low level of promoter bivalency (H3K4me3/H3K27me3) [18, 19], and a low level of H3K9me2 [20], in comparison to conventional culture (in fetal bovine serum and LIF) or primed pluripotent states such as epiblast-derived stem cells. Moreover, female ESCs and iPSCs, which exhibit XaXa state for X chromosome, strongly downregulate genomic DNA methylation [16].

CpG methylation is of great importance in controlling epigenetic gene silencing and genome stability maintenance. In mammals, CpG methylation state is regulated by the coordination of three CpG DNA methyltransferases (DNMTs), namely, DNMT1, DNMT3a, and DNMT3b [21, 22]. In mice, deletion of* Dnmt1* and* Dnmt3b* leads to embryonic lethality, and deletion of* Dnmt3a* results in postnatal lethality [21, 22], indicating their crucial roles in development. However, ESCs can still maintain chromosomal stability and stemness properties in the absence of CpG methylation induced by triple knockout of these three DNMTs* in vitro* [23]. In addition, passive demethylation induced by 2i treatment results in suppression of DNMT3a, DNMT3b, and DNMT3L and the hypomethylated state in ESCs [15, 17, 24].

Besides, Ten-Eleven Translocation (TET) family proteins are also important regulators of DNA methylation through the conversion of 5-methylcytosine (5mC) to 5-hydroxymethylcytosine (5hmC) of DNA [25]. Both TET1 and TET2 are able to convert 5mC to 5hmC, yet their regulations of 5hmC in mouse ESCs are distinct, for TET1 functions primarily at transcriptional start sites, whereas TET2 decrease 5hmC in gene bodies [26]. Following ESC differentiation, both TET1 and TET2 levels will be downregulated; conversely, when fibroblasts are reprogrammed into induced pluripotent stem cells (iPSCs), TET1 and TET2 levels will increase [27]. These findings indicate that TET1 and TET2 are closely associated with the pluripotent state. Interestingly, it is suggested that OCT4, which is sufficient in inducing pluripotency of somatic cells to express other reprogramming factors, could be replaced by TET1 in reprogramming cocktail [28].

In addition, polycomb complexes and mixed linage leukemia (MLL) mediated histone modifications are also important. It is reported that polycomb complexes regulate hundreds of genes in mammals and insects [29]. In stem cells, they are transcriptional regulators through epigenetic modifications, controlling stem cell identity and differentiation. Among the polycomb repressive complexes (PRCs), PRC1 and PRC2 are focused on by recent studies [30]. PRC2 is an H3K9me3 binder maintaining heterochromatin and is essentially needed for H3K7me3 levels [31, 32]. It was reported that SUZ12, which formed PRC2/3 with EZH2 and EED, was required for cell proliferation and for the activity as well as stability of PRC2 or PRC3 complexes. Interestingly, SUZ12 deficiency resulted in the loss of dimethylation and trimethylation of H3K27 [33, 34]. In addition,* Eed* deficiency in ESCs led to dramatically reduced EZH2 levels and decreased histone H3K27 methylation levels [35, 36]. Moreover, absenceof* Ezh2* expression resulted in abrogation of paternal genomic contraction and activation of several* cis* genes in extraembryonic tissues after implantation [36]. In particular,* Eed*-knockout ESCs lack all H3M27 methylations [35], while SUZ12 knockout ESCs maintain H3K27me1 in part [34], suggesting EED might function upstream PRC2 as well as serving as a part of PRC2.

MLL family contains a large number of members, among which MLL1 and MLL2 are most investigated in terms of pluripotency. MLL1 mainly works to catalyze the monomethylation of H3K4, whereas MLL2 functions primarily to regulate trimethylation of H3K4. Thus, MLL1 and MLL2 affect the self-renewal and pluripotency profile of ESCs by regulating the methylation states of H3K4 [37, 38]. It is reported that in MLL1-null epiblast stem cells, cell reprogramming will be launched and the cells will restore a naïve pluripotency state [39]. Moreover, in ESCs, deletion of MLL2 will lead to increased apoptosis profile as well as abnormal differentiation [40].

## 3. Primordial Germ Cells

During the development of mouse embryo, a cluster of about 40 cells appears at the base of the allantois at E7.25, which is positive for alkaline phosphatase and PR domain containing 1, with ZNF domain (Prdm1), and is considered as the precursors of PGCs [41, 42]. Soon after, these cells exhibit polarized morphology. At E7.75, they migrate to the developing hindgut and colonize the genital ridges at about E10.5 [42, 43].

### 3.1. Molecular Control of PGCs

During this process, PGCs proliferate robustly and express their specific genes. Among the regulators in this process, (PR domain containing protein 1) Prdm1 and Prdm14 are the two most important proteins for PGC specification. Prdm1 is initially identified as a transcriptional repressor. It could interact with various epigenetic regulators in a context-dependent manner primarily to repress the transcription of somatic cell genes and stimulate the expression of PGC specific genes [19, 44]. Prdm14 also functions as a transcriptional regulator and is necessary for PGC specification [45]. Prdm1 and Prdm14 are also crucial for epigenetic regulations of PGC, regulating the expression of downstream genes synergistically, thus promoting the pluripotency of PGCs [43, 46–48].

### 3.2. Epigenetic Reprogramming in PGCs

During the process of PGC migration and proliferation, the DNA methylation levels should be controlled accurately to guarantee proper erasure of parental genomic imprints [49]. PGCs are induced in the epiblast at around E6.5 and first arise as a population of approximately 40 cells at E7.25 in the proximal epiblast; at E9.5, a group of approximately 200 PGCs begins to migrate through the hindgut endoderm and arrives at the gonadal anlagen at about E10.5–E11.5 [50]. During this process, the overall methylation level at CpG dinucleotides decreases gradually, with the bulk of methylation erasure occurring prior to E9.5. Moreover, this demethylation process is unidirectional, with no* de novo* methylation process between E6.5 and E13.5 [51]. At E6.25, signals mediated by bone morphogenetic protein (BMP) result in PGC specification, which is initiated by assigning some epiblast cells to become PGCs [52]. During PGC specification, DNA methylation reprogramming for totipotency is activated. A recent study constructed DNA methylation maps of murine PGC-like cells (PGCLCs), induced from ESC-derived epiblast-like cells (EpiLCs), as a model of PGC specification. ESCs reorganize methylome to form EpiLCs through hypomethylated domains at pluripotency regulator regions, whereas PGCLCs constantly dilute the EpiLC methylome by accumulating H3K27me3 around developmental regulators [53]. Moreover, several studies from different groups reported replication-coupled passive mechanism for the erasure of DNA methylation. During the period of genome-wide DNA demethylation, PGCs bear little* de novo* or maintenance DNA methylation potential, erase genome imprints with varying rates, and show rapid cell cycle, with no apparent major chromatin alteration [51, 54, 55]. The status of reprogramming into naïve pluripotency needs the interwork of different factors [56]. Among them, TET1- and TET2-mediated 5mC-to-5hmC conversion is important to modulate DNA methylation levels and drive comprehensive reprogramming of PGCs [57–59]. It was reported that the loss of TET1 is harmful to germ cell formation in embryo and would cause infertility in both females and males [60, 61]. Another factor affecting DNA methylation is STELLA, which is the first marker associated with epigenetic modifications during the development of PGCs, with an increased expression level at E7.0–E7.5 [62]. It was reported that STELLA was indispensable for maintaining the methylation state of PGCs and was required for the maintenance of maternal genome methylation in the zygotes [63]. UHRF1, which encodes NP95 protein, is essential for maintaining local and global DNA methylation and repressing transcription of retrotransposons and imprinted genes [64]. DNMT3a and DNMT3b are indispensable for* de novo* methylation and thus for mouse development [21]. In wild-type PGCs, both UHRF1 and DNMT3a/DNMT3b are repressed [19, 65], resulting in lack of* de novo* and maintenance mechanisms of DNA methylation, which is considered to contribute to global DNA demethylation. In consistency with this argument, genomic DNA methylation is erased in a replication-coupled manner [51, 54, 55, 66]. In addition, PRDM14, which is exclusively expressed in pluripotent cells and germ cell linages, is crucial for the reacquisition of potential pluripotency and epigenetic reprogramming. In PRDM14 knockout embryos, these two events fail to occur even in the presence of PRDM1. PRDM14 knockout mice lack germ cells and are thus sterile, with a defect in genome-wide epigenetic reprogramming and shifted ratios of H3K9me2 and H3K27me3 in the mutant PGCs [45]. Moreover, PRDM14 knockout or knockdown studies also implicated that PRDM14 is also involved in hypomethylated states in naïve ESCs by repressing DNMT3a/DNMT3b [15, 17, 24].

The histone modifications of PGCs are primarily reflected by the change of H3K9me2 and H3K27me3, which are the two unique PGC histone modification patterns important for the proper development of PGCs. During the induction of mouse ESCs to EpiLCs and to PGCLCs* in vitro*, it was demonstrated that EpiLCs contained low H3K27me3 levels in bivalent gene promoters, whereas PGCLCs lose H3K4me3 from bivalent genes with a concomitant increase of H3K27me3. Moreover, PGCLCs lose H3K9me2 progressively which led to changes in nuclear architecture, ensuring normal development of PGCs [19, 67, 68]. In PGCs, H3K9me2 is inhibited at E7.25, whereas H3K27me3 increases at E8.25 [69], which indicates that H3K9me2 lies at the upstream of histone modifications. Moreover, it seems that histones do not work alone but will interact with various factors to accomplish the genome-wide demethylation in PGCs. It has already been proved that H3K9me2 could bind STELLA directly, and inhibition of H3K9me2 will result in failure of STELLA recruitment and decreased DNA methylation levels [70]. In addition, increased H3K27me3 levels will increase the level of Ezh2, which is important in pluripotency maintenance as well as demethylation regulation [71].

## 4. Spermatogenesis

### 4.1. The Process of Spermatogenesis

Spermatogenesis is a complicated process. During this process, spermatogonial stem cells (SSCs) launch various regulating mechanisms to accomplish a delicate balance between self-renewal and differentiation [72]. The most primitive SSCs are called A-single spermatogonia, which were located at the basement membrane [73]. A-paired spermatogonia, which contain two differentiating spermatogonia connected by an intercellular bridge, would be generated from A-single spermatogonia because of incomplete cytokinesis. Then, the A-paired spermatogonia continue to divide and generate chains of 4, 8, 16, and sometimes 32 cells, called A-aligned spermatogonia, which finally generate type B spermatogonia [74–76].

The final stage of SSC mitotic division generates type B spermatogonia, which finally divides into preleptotene spermatocytes, reflecting the beginning of meiosis. After two rounds of meiosis, diploid spermatogonia will differentiate into haploid round spermatids. Finally, the round spermatids undergo spermiogenesis, after which their shape elongates and undergoes cytological changes, and mature spermatids will be generated at last [73].

### 4.2. Epigenetic Regulations during Spermatid Development

The differentiation of SSCs to advanced spermatogonial cells cannot be accomplished without proper histone regulations. In fact, canonical histone synthesis occurs only in S-phase but will play a role effectively throughout the whole cell cycle [77]. Spermatogonia maintain pluripotent state during stages of A-single to A-aligned stages. At this stage, monomethylated H3K27 and H4K20 are completely lacking and with little monomethylated H3K9 [78]. In spermatogonia, many regions show a stage-specific differential methylation pattern in and around loci which are important for spermatogenesis and stem cell functions [79]. Moreover, spermatogenesis could be launched without changing DNA methylation pattern and instead associated with transcription of certain DNA-methylated promoters [80]. The spermatocytes from Prdm9-null mice express some genes specific to autosomes, whereas the genes which should be expressed during meiosis are repressed [81]. It was also reported that double mutations of Suv39h1 and Suv39h2, which are both trimethyltransferase genes of H3K9, will lead to nonhomologous chromosome associations [82]. Therefore, it is possible that H3K4me3, mediated by Prdm9 and H3K9, play critical roles during the association of homologous associations.

### 4.3. Epigenetic Regulations in Spermiogenesis

During spermiogenesis, the expression of histone variants is universal and at a large scale. The histone variants include H1T, H1T2, H1LS1, TH2A, TH2b, H3.3, and H3.5. They work cooperatively and are indispensable for meiosis progression as well as the formation of mature sperms [81, 83].

H1T plays important roles in the initiation of meiosis. Compared with other H1 histones, H1T binds much less tightly to H1 depleted oligonucleosomes, which help to maintain a relatively loose chromosome configuration, guaranteeing the initiation of meiosis [84, 85]. In addition to this, H1T is exclusively detectable in mid- to late-pachytene spermatocytes [85, 86]. Another crucial histone in meiosis is TH2B, which show high expression levels from leptotene spermatocytes starting at P10 [87]. Interestingly, both TH2A and TH2B genes are located in chromosome 17 and share a common promoter, suggesting that they may have redundant functions in germ cells [88, 89].

During spermiogenesis, the majority of the core histones will be replaced, first by transition proteins and then by protamines, resulting in chromatin hypercompaction [90]. The histone-protamine transition is a hallmark of epigenetic regulation in the male germline development. During this process, hyperacetylation of histone H4 and monoubiquitination of H2A occur, which are suggested for better enzyme access as well as chromatin remodelers [91], and are demonstrated to be an essential feature—but not the only inducer—of histone-protamine transition [92]. Appropriate ubiquitination is another factor needed in histone-protamine transition, and it has been demonstrated that RNF8 is crucial in mediating H2A/H2B ubiquitination and for the normal replacement of histones with nucleoprotamines during spermiogenesis [93]. In addition, methylation is also of importance during spermiogenesis, of which the H3K79 methylation is indicated to play a critical role during histone replacement [94, 95].

During spermiogenesis, the nuclei of haploid spermatids will be condensed through the replacement of nucleosomes with protamines in a genome-wide fashion. Nevertheless, a fraction of nucleosomes remains associated with sperm genome. The biological significance of this phenomenon is still elusive and has been actively debated [96]. For example, while some studies reported that the remained nucleosomes are preferentially enriched at promoter regions and exons in mouse sperms [97] and at loci of developmental importance in human sperms [98], other studies demonstrated that the retained nucleosomes are significantly enriched within distal gene-poor regions and are significantly depleted in promoters of developmental importance [99, 100]. Besides, an evenly distributed form of nucleosomes alongside the whole genome of human sperm with only a small proportion of enrichment within the transcriptional start sites was also observed [101].

During spermatid elongation, the histone variants work cooperatively, thus guaranteeing the production of mature sperm with normal functions. For example, H1T2 is critical for the formation of acrosomes, whose deletion will result in a greatly reduced fertility because of abnormal spermatid elongation as well as defective DNA condensation [102, 103]. Moreover, H1SL1, H3.3, and H3.5 could all promote the condensation of chromosomes, ensuring the regular exchange of histones and protamines [81, 83, 104].

## 5. Conclusion

As the transmission mediator of hereditary information, gametes have been attracting the attention of scientists all these years. In particular, epigenetic regulation patterns are investigated extensively and much progress has been made in this field. Now, it is more and more clear about the epigenetic modification controls during the process of ESCs to PGCs, yet the regulation mechanisms during spermatogenesis are still elusive. Nevertheless, our understanding of the epigenetic mechanisms during the whole process of ESC development and spermatogenesis is still preliminary to some extent, and we know it for sure that a deeper understanding about these regulations will contribute greatly to the study of spermatogenesis. Besides, we should note that PGC differentiation from ESCs is only an* in vitro* reconstitution system, which is used as a platform for reconstitution of male or female haploid germ cell development [105–109] and for epigenome studies in germ cell specification [19, 66].
